# Efficacy of endocervical curettage and CA-125 measurement in endometrial serous carcinoma: A case series and literature review

**DOI:** 10.4274/tjod.69926

**Published:** 2015-09-15

**Authors:** Ahmet Cem İyibozkurt, Murat Doğan, Ercan Baştu, Hamdullah Sözen, Doğan Vatansever, Samet Topuz, Sinan Berkman

**Affiliations:** 1 İstanbul University İstanbul Faculty of Medicine, Department of Obstetrics and Gynecology, İstanbul, Turkey

**Keywords:** CA-125, Endometrial cancer, endocervical curettage, endometrial serous carcinoma

## Abstract

**Objective:**

This is a case series and literature review of patients with endometrial serous carcinoma (ESC) in which endocervical curettage (ECC) and CA-125 measurement were utilized as a diagnostic procedure in preoperative staging.

**Materials and Methods:**

The patients were treated in the gynecologic oncology clinic of İstanbul University Faculty of Medicine between January 2005, and January 2015. A total of 37 patients were included in the final analysis.

**Results:**

ECC accurately predicted ESC in 22 patients (59.5%). The mean pre-operative serum CA-125 level was 73.24±3.30 IU/mL; pre-operative serum CA-125 levels were elevated above 35 IU/mL in 25 patients (69%).

**Conclusion:**

ECC is an acceptable diagnostic tool to predict the presence or absence of cervical involvement in endometrial cancer. On the other hand, its accuracy in specific subgroups requires further analysis in carefully designed prospective studies. Furthermore, pre-operative serum CA-125 levels may be important for management and counseling in the subgroup of women with ESC.

## INTRODUCTION

Endometrial carcinoma is the most common gynecologic malignancy^(1)^. It includes carcinomas composed of glands that look like endometrium (endometrioid subtype)^(2)^ as well as differentiated epithelial neoplasms, which usually appear in the extended Müllerian system^(3,4)^. Endometrial endometrioid carcinoma is the most frequent subtype of endometrial carcinoma; endometrial serous carcinoma (ESC) is a well-recognized but rare subtype that is usually associated with aggressive behavior and poor clinical survival^(3,5,6,7)^.

Endocervical curettage (ECC) has been commonly used to evaluate cervical involvement, but its accuracy remains debatable^(8,9,10,11,12,13,14)^. As a result, pathologic diagnosis may not be accurate due to the presence of metaplastic changes that may hide or look like a malignancy. This is a serious concern, especially for postmenopausal patients, in which endometrial cancers are generally an important diagnostic consideration.

Elevated levels of tumor-associated antigen CA-125 were first described in patients with recurrent and advanced endometrial cancer by Niloff et al.^(15)^ in 1984. Several studies have since shown that elevated pre-operative serum CA-125 correlates with extra-uterine tumor spread^(16,17,18,19)^. However, all these studies included different types of histology. To our knowledge, none of the previous studies in the literature have evaluated the potential clinical implications of pre-operative serum CA-125 solely in patients diagnosed as having ESC.

This is a case series and literature review of patients with ESC in which ECC and CA-125 measurements were utilized as diagnostic procedures in preoperative staging. We hypothesized that these tools would have an impact on prognosis in patients with ESC.

## MATERIAL AND METHODS

For this retrospective study, consecutive patients with endometrial cancer (n=754) treated in the gynecologic oncology clinic of İstanbul University School of Medicine between January 2005, and January 2015 were reviewed. Forty-three patients with histologically-verified ESC who underwent primary surgery at our institution were identified. We excluded 6 patients from the analysis because of incomplete data. A total of 37 patients were included in the final analysis. All clinical records were reviewed for demographics, treatment details, and outcomes. The study protocol was approved by the Ethics Committee of İstanbul University and informed consent was waived due to the retrospective nature of the study.

In every case, the surgery was performed by a gynecologic oncologist. Staging surgery was in accordance with the International Federation of Gynecology and Obstetrics (FIGO) adopted system^(20)^ and included peritoneal washing for cytology, total abdominal hysterectomy, bilateral salpingo-oophorectomy, pelvic and para-aortic lymphadenectomy.

CA-125 levels were pre-operatively measured in all 37 patients. In each case, the diagnosis was confirmed by a specialist gynecologic pathologist following post-surgery pathology review. Preoperative histopathologic assessment of cervical involvement was also performed through ECC in all 37 patients.

Surgical pathologic information was gathered from the pathology reports, which included histologic tumor type, grade, FIGO stage, and tumor features, such as lymphovascular invasion (LVI).

Continuous variables were described as mean ± SD and categorical data were expressed in number and percentage.

## RESULTS

The mean age of the patients was 63.20±10.72 years (range, 38 to 85 years). ECC accurately predicted ESC in 22 patients (59.5%). The mean pre-operative serum CA-125 level was 73.24±3.30 IU/mL; pre-operative serum CA-125 levels were elevated above 35 IU/mL in 25 patients (69%).

Post-operative pathology revealed endometrioid carcinoma in 9 patients (24.3%) (grade 1 in 1 patient, grade 2/3 in 8 patients), clear cell carcinoma in 3 patients (8.1%), mixed epithelial and stromal tumor in 2 patients (5.4%), malignant mixed Müllerian tumor, also known as carcinosarcoma, in 1 patient (2.7%).

Of the 25 patients (67.6%) who underwent lymph node dissection, pelvic lymph node metastases were detected in 2 patients, and LVI was present in 50% of patients.

## DISCUSSION

When the diagnostic value of ECC in predicting ESC was analyzed in our study, ECC accurately predicted ESC in nearly 60% of cases. Misclassification occurred in approximately 40% of all cases. Positive predictive values of ECC varied in previous studies (15.1-62.5%) in patients with endometrial cancer^(10,12,21,22,23)^. These differences can partly be explained by heterogeneity in patient selection and differences in the curettage assessment. It is also important to note that no previous studies have solely focused on the subgroup of patients with ESC.

The tumor antigen, CA-125, was first introduced by Bast et al.^(24)^ in 1981. Since then, it has been evaluated for ovarian cancer screening,^(25,26,27)^. diagnosis,^(28,29,30)^ and post-treatment monitoring^(31,32,33,34)^. Thus far, the literature does not support the routine clinical use of CA-125. However, it helps in diagnosis and has become an accepted method of monitoring response to treatment, as well as disease recurrence and progression. In endometrial cancer, the use of pre-operative CA-125 has been evaluated in several studies. In most of these studies, elevation of CA-125 correlated to the presence of extra-uterine disease^(18,35,36,37,38,39)^. However, none of these studies performed a subgroup analysis of ESC, probably because of the limited number of patients. To our knowledge, this present study is the first to evaluate the role of pre-operative serum CA-125 exclusively in patients with confirmed ESC. Our study revealed an elevation of CA-125 in nearly 70% of patients with ESC, which is in line with previous findings.

Lesions in this morphologic range have to be accurately diagnosed because the prognostic profile of ESC is distinct. Therefore, an additional method of assessing for the presence of extra-uterine disease such as serum CA-125 seems a reasonable pre-operative approach and would be of benefit in pathologic diagnosis and clinical management. Assessment of HER2 immunohistochemistry was also recently recommended in ESC because of the significant heterogeneity of HER2 protein expression^(40)^. However, further studies are needed to confirm its efficacy for routine clinical use.

The major limitation of this study is the small number of patients involved; however, this is accounted for because ESC is a rare subtype and is much less common than its endometrial counterparts.

## CONCLUSION

ECC is an acceptable diagnostic tool to predict the presence or absence of cervical involvement in endometrial cancer. On the other hand, its accuracy in specific subgroups requires further analysis in carefully designed prospective studies. This study contributes to the growing literature on the use of pre-operative serum CA-125 in patients with endometrial cancer. Moreover, it suggests that pre-operative serum CA-125 levels may be important for management and counseling in the subgroup of women with ESC.
